# Subnuclear distribution of proteins: Links with genome architecture

**DOI:** 10.1080/19491034.2017.1361578

**Published:** 2017-09-14

**Authors:** Fouziya R. Shah, Younus A. Bhat, Ajazul H. Wani

**Affiliations:** a Biotechnology, School of Biological Sciences, University of Kashmir, Srinagar, India

**Keywords:** cajal bodies, chromatin architecture, chromosome conformation capture, insulator speckles, microscopy, polycomb clusters, subnuclear distribution of proteins

## Abstract

Metazoan genomes have a hierarchal 3-dimensional (3D) organization scaling from nucleosomes, loops, topologically associating domains (TADs), compartments, to chromosome territories. The 3D organization of genome has been linked with development, differentiation and disease. However, the principles governing the 3D chromatin architecture are just beginning to get unraveled. The nucleus has very high concentration of proteins and these proteins are either diffusely distributed throughout the nucleus, or aggregated in the form of foci/bodies/clusters/speckles or in combination of both. Several evidences suggest that the distribution of proteins within the nuclear space is linked to the organization and function of genome. Here, we describe advances made in understanding the relationship between subnuclear distribution of proteins and genome architecture.

## Introduction

Folding of long genome into the specific 3-dimensional (3D) conformation within the tiny cell nucleus is attained with the help of proteins associated with it. Some proteins enzymatically modify the chromatin and facilitate acquisition of the required conformation. Others physically compact it by winding the DNA around them, like histones, or act as physical links via protein-protein interactions between distant genomic regions and thus help in folding the genome within the nucleus. Interactions among proteins bound at different sites along the genome can result in association of proteins into subnuclear foci/clusters. However, it is also possible that some subnuclear protein clusters are devoid of DNA and act as storage or sequestration sites of proteins and modulate organization of genome indirectly. Unraveling how organization of nuclear proteins is related to the genome organization will provide novel insights into the principles governing the 3D organization of genome.

Different cell types of an organism possess the same DNA but during development and differentiation their genomes get biochemically, structurally and organizationally modified to attain cell type specific gene expression. Hence, organization of genome is different in different cell types. Analogous to the protein structure-function relationship, 3-dimensional organization of genome has been linked to genome function and overall development of an organism.^1-4^

Here, we will first briefly highlight different features of chromatin organization by using parallel sets of evidence from chromosome conformation capture and microscopy based experiments followed by brief description of subnuclear distribution of proteins and then elaborate the links between the two.

### Chromatin organization

**Figure 1.**
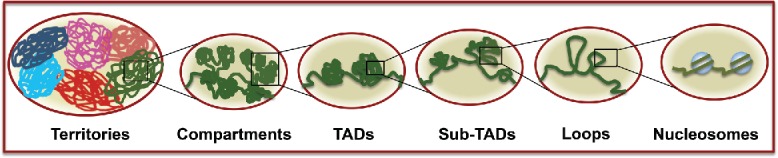
Genome organization: Eukaryotic genomes have hierarchal organization varying from nucleosomes, loops, sub-topologically associating domain (sub-TADs), topologically associating domains (TADs), compartments to chromosome territories.

Organization of metazoan genomes scales from nucleosomes, loops, TADs to chromosome territories (Fig. 1) and application of various biochemical, genomic, imaging and polymer modeling methods have revealed many intricate details of genome organization. Genomic contact maps obtained from chromosome conformation capture based methods have revealed the topological features of genome organization.^5^ From these studies TADs have emerged as units of high order eukaryotic genome organization.^6^ TADs are formed by establishment of contacts within consecutive DNA sequences resulting in formation of a folded domain. Between 2 successive TADs lie DNA elements with fewer contacts called boundary regions. TADs have been observed in different organisms from yeast to humans.^7-12^ Evidences supporting the existence of TADs have also come from microscopy studies, where DNA sequences belonging to the same TAD were found to lie closer to each other than sequences present in 2 different TADs.^13^ Visual demonstration of TADs came from a study on *Drosophila* larvae salivary gland polytene chromosomes, in which TADs and boundaries were shown to correspond to bands and interbands seen under microscope.^14^ TADs have also emerged as functional domains of genome. TADs can provide the confinement within which promoters interact with their respective enhancers. Clustering of silenced or active genes on X-chromosome also correlates with TADs.^15^. Besides the transcriptional activity, TADs also correlate with the replication domains as TAD boundaries coincide with replication domain boundaries.^16^

Organization of genome at genomic and spatial scales both beyond and below the level of TADs have been studied. An elegant *in situ* Hi-C study showed that TADs are composed of sub-TADs which have an average size of 185 kb.^9^ 5C analysis of different genomic loci at early developmental stages have identified sub-TADs some of which get rearranged during differentiation of embryonic stem cells (ESCs) to neuronal progenitor cells (NPCs).^10^^,^^17^ Recently Kundu et al., identified sub-mega base, polycomb group (PcG) protein repressed discrete domains distinct from TADs and the organization of these PcG domains changed upon differentiation of ESCs to NPCs.^18^ In an elegant *in situ* hybridization coupled to super-resolution microscopy study different epigenetic chromatin states were found to have distinct folding. In comparison to inactive and active domains, PcG-repressed domains were found to have the most dense packing with high degree of intermixing.^19^

At the higher level, TADs are further organized into spatial compartments. Hi-C studies have shown that active and inactive TADs cluster in different compartments and sub-compartments.^8^^,^^9^^,^^20^ A multiplexed fluorescence in situ hybridization (FISH) analysis demonstrated that TADs of individual autosomes and X chromosome are spatially arranged into different compartments and these compartments were observed in most of the individual cells analyzed.^21^ These studies suggest that compartments are stable physical structures separating the inactive and active TADs of a chromosome. At the chromosome level of organization a territorial model has been proposed according to which chromosomes occupy distinct regions within the nuclear space called chromosome territories (CTs). Several evidences suggest that CTs are an important and prominent feature of genome organization.^22-25^ However, excursions of chromatin regions from CT causing intermingling of chromosomes have been observed. In addition to the visual evidences provided by imaging studies, the 3C based studies have also independently supported the existence of CTs from yeast to mammals.^8^^,^^26^^,^^27^

So far we briefly highlighted the main features of the 3D genome architecture. Although we have gained a wealth of information about different features of genome organization, the principles governing the 3D organization of genome within the nuclear space are just beginning to get unraveled. Numerous studies suggest that nuclear proteins can be key players in shaping the genome architecture. Starting at the genomic scale of 146 bp, histone proteins comprising nucleosomes provide the very first level of packaging. At higher level there are loops between enhancers and promoters that are mediated by transcription factors, different TADs are enriched in different chromatin associated protein, boundaries separating TADs are bound by insulator proteins and lamins (long with other proteins) are involved in radial arrangement of chromosomes. Hence, studies investigating mechanistic details by which nuclear proteins stabilize and regulate the dynamics of genome organization at multiple genomic and spatial scales are in high demand. In this review we will describe the relationship between genome organization and sub-nuclear distribution of proteins.

### Subnuclear distribution of proteins

**Figure 2.**
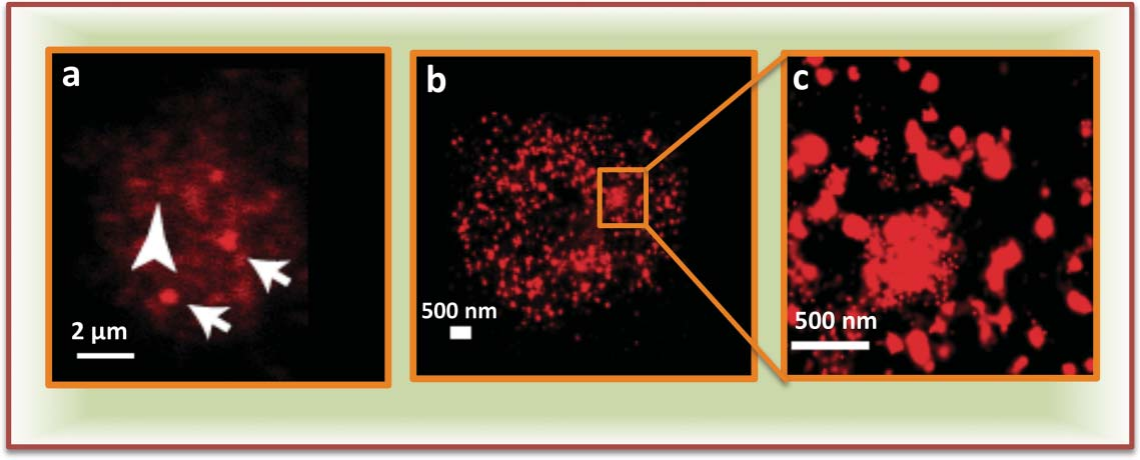
Subnuclear distribution of proteins: (a) A confocal image showing aggregated (arrows) and diffused (arrow head) fractions of a polycomb protein, polyhomeotic. (b) Super-resolution image showing the distribution of same protein in same cell type. (c) Zoom in from b showing nano-scale subnuclear clusters (modified from Wani et al., 2016^29^).

Microscopy based studies have revealed enormous information about the subcellular distribution of proteins. Immunofluorescence imaging of the nuclear proteins in general shows either a diffuse distribution, an aggregated/punctate distribution, or a combination of both diffuse and aggregated (Fig 2). Before describing the “diffused” and “aggregated” fractions it is important to note that these definitions are relative and depend on the resolution of the microscope. Macromolecules even if aggregated/clustered will appear diffused if their size is below the diffraction range of light used to image.^28^^,^^29^

**Figure 3.**
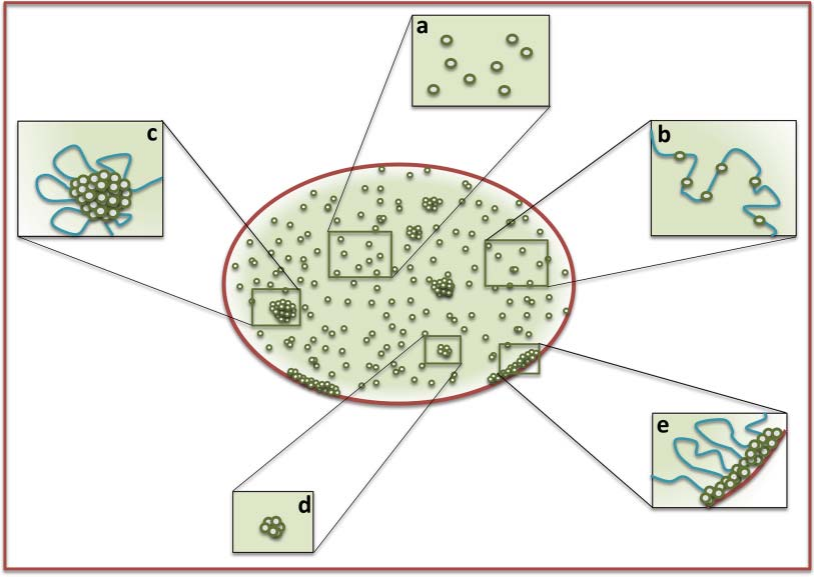
Schematic showing sub-fractions of diffused and aggregated fractions: Diffused fraction of protein can be either freely diffusing (a) or bound to chromatin (b). Aggregated fraction can also be either bound to chromatin (c) or floating freely in the nucleoplasm (d). Some of the aggregated fraction can be bound to nuclear membrane and chromatin (e).

The “diffused fraction” in the nucleus is likely composed of proteins either in monomeric or oligomeric form with dimensions escaping the resolution limit of microscope used for imaging. This fraction can be further divided into 2 fractions; proteins which are either randomly diffusing within the nucleus and proteins bound to chromatin (Fig 3). Examples of the diffused fraction include transcription factors, enzymes and chromatin remodeling factors either free in the nucleoplasm or transiently binding to the chromatin and other proteins not related to chromatin dynamics but floating within the nucleus.

The “aggregated fraction” appears as clusters/foci/bodies. These structures comprise of either proteins only, or proteins and RNA, or proteins and DNA, or proteins, DNA and RNA. They can be either small and randomly diffusing or they can form well-assembled compartments within the nucleus like nucleolus. This fraction has been studied more thoroughly than the diffused fraction and includes different types of bodies/clusters like cajal bodies, promyelotic leukemia (PML) bodies, PcG clusters/bodies, insulator bodies/speckles etc. These are membrane-less, dynamic structures, working as open systems slightly denser than surrounding nucleoplasm as their components readily exchange with freely diffusing molecules in the nucleoplasm.^30^^,^^31^ Fluorescence recovery after photobleaching (FRAP) experiments of paraspeckles have shown that the paraspeckle core proteins exchange rapidly with nucleoplasmic pool with a t_1/2_ of the order of seconds.^30^ Similarly, In case of PcG clusters, PcG proteins were found to exchange between cluster and soluble pool and the exchange rates were found to be different between interphase and mitotic cell.^32^

Different models have been proposed for the formation of *nuclear bodies* and have been reviewed before.^30^ Cajal bodies seem to form stochastically from different components while as assembly of paraspeckles and nucleoli starts from specific RNA transcripts acting as seeds. In contrast, assembly of *Drosophila* HLBs appears to start from protein seeds involving Mxc and FLASH and follows a hierarchical assembly.^33^ In addition to these models, assembly of nuclear bodies though phase transition has been proposed.^34^ Phase transitions have strong dependence on concentration of different molecular species and temperature. Assembly of Ddx4 protein into liquid like droplets showed dependence on expression level of protein, ionic strength and temperature in a manner similar to that of a phase transition phenomena.^35^ Also incase of *Caenorhabditis elegans* embryos the size of nucleoli was found to strongly correlate with the concentration of different nucleaolar components, indicating the possibility of phase transition type of assembly. Recently, Larson et al., showed that heterochromatin protein (HP1α) forms phase separated liquid droplets and these droplets may sequester chromatin and establish heterochromatin.^36^ However, given the dynamic and heterogeneous nature of nuclear bodies it is very challenging to determine their mechanism of assembly.

The nuclear bodies appear to be stabilized by hubs of intermolecular interactions, including protein-protein, protein-RNA and protein-DNA interactions. Analysis of about 3000 proteins constituting different nuclear compartments like Cajal bodies, nucleolus, promyelotic leukemia bodies etc. showed that most of these proteins have disordered domains, which enhance their ability to promiscuously interact and bind with other proteins. In line with this, protein-protein interaction networks of these nuclear compartments were found to have more interaction hubs than non-nuclear proteins.^37^

Nuclear bodies have been described as multifunctional compartments.^29^^,^^30^ They are involved in diverse functions varying form rRNA biogenesis, processing and modifications of non-coding as well as coding RNAs, telomere maintenance, ribosome assembly, cell cycle progression, transcription, DNA repair, genome stability, apoptosis and stress. These functions of NBs have been extensively reviewed before.^30^^,^^31^ Our aim here is to specifically describe the relationships between nuclear bodies/clusters/speckles and genome organization. In the following sections we will explain the connection between subnuclear protein distribution and genome architecture by highlighting the more recent research in the field.

### And the links

We broadly classified the distribution of the nuclear proteins into the “diffuse” and “aggregated” fractions. First we will explain how the “diffused” fraction of proteins can affect the genome organization followed by the role of different nuclear bodies/cluster/speckles, falling under “aggregated” fraction in shaping 3D genome organization.

### Diffused fraction and chromatin organization

The diffused fraction can be divided into 2 sub-fractions “freely” diffusing molecules and molecules “bound” to the chromatin (Fig. 3). Freely diffusing proteins contribute to the rheological (like viscosity) and crowding properties of the nucleoplasm. Given the viscous nature of the nucleoplasm it can impede the diffusion of large macromolecular assemblies. A study estimated that forces in the range of few piconewton are required to move the nuclear bodies within the nucleoplasm.^38^ Intuitively, this property of nucleoplasm should hinder the diffusion of chromatin domains and decrease the probability of long-range chromatin interactions. However, on the other hand some studies suggest that molecular crowding leads to compaction of chromatin. This effect has been explained on the basis of depletion forces, which can arise because of the differential size of protein molecules and association of larger chromatin domains.^39^ The latter causes an increase in the volume available to smaller molecules due to the overlap between exclusion volume of chromatin domains upon self-association. The compaction also seems to be entropically favored as it appears to increase the overall entropy of the system.^40^ Although there is loss of mixing entropy upon compaction/association, due to increase in volume available to smaller molecules their configurational freedom increases resulting in net increase of entropy. Effects of changing nuclear crowding on chromatin organization have been studied by treating cells with different agents like sucrose or polyvinylpyrrolidone (PVP). Upon treatment of cells with medium containing high concentrations of sucrose chromatin gets reorganized into dense staining domains. Interestingly, these effects on chromatin compaction were found to be reversible when cells were transferred into normal medium.^41^ Similar compaction effects were observed when cells were treated with PVP or Dextran, but the experiments were done with permeablized cells.^41^^,^^42^ Although from these studies observed chromatin reorganization seems to arise because of molecular crowding, the hypertonic treatments can affect ionic strength and can alter the binding affinities of proteins with DNA or induce post-translational modifications (PTMs),^43^ which in turn can result in changes in chromatin organization. Hence, further studies are needed to dissect specifically the contribution of crowding effects on the organization of chromatin.

The “bound” component of the diffused fraction includes proteins, which are bound to chromatin at distinct sites in their monomeric or oligomeric form. These can be DNA binding proteins like transcription factors, histone modifying enzymes, ATP-dependent chromatin remodeling factors etc. These factors can spend different durations on the chromatin before falling off and hence can shuttle between bound and free fractions. Their distribution between these fractions can also get altered upon external signals, cell cycle stage etc. Chromatin immunoprecipitation coupled to genome wide sequencing studies have provided enormous information about binding profiles of various chromatin associated proteins along the genome. In general, binding of proteins to the chromatin will increase its stiffness and alter its persistence length which in turn can affect the folding of chromatin, and hence its organization. Discussing the role of different proteins like individual transcription factors, histone-modifying enzymes etc. falling in this fraction, in modulating chromatin structure is beyond the scope of this review.

### Aggregated fraction and chromatin organization

The “aggregated fraction” as we defined above comprises of proteins which appear as clusters/bodies/foci/speckles. In the following sections we will discuss the relationship between some of these subnuclear structures with genome organization.

### Cajal bodies

Cajal bodies are one of the prominent subnuclear structures besides the nucleolus and have been discovered more than a century ago. They appear in shape as round to irregular foci usually found in-between chromosome territories. Their observed size varies from 0.5–2 μm, and their number per cell also varies in different organisms, at different stages of development (more at earlier stages of development), phases of cell cycle and with metabolic state of the cell.^44^ Cajal bodies are composed of many proteins and RNAs forming a complex ribonucleo-protein assembly (Table 1). Coilin is a typical protein of cajal bodies and has been used as a marker to track cajal bodies. Cajal body proteins, like other nuclear component proteins have disordered domains and have more tendency to form protein-protein interaction networks (PPINs) compared with non-nuclear proteins.^37^ The identified proteome of cajal bodies has expanded since their initial discovery and many of the proteins are shared between cajal bodies and other subnuclear structures like nucleolus, histone locus bodies (HLB), and promyelocytic leukemia (PML) bodies.^44^ The RNA fraction of cajal bodies includes small nuclear RNAs, small nucleolar RNAs and cajal body specific sca-RNAs. Cajal bodies are involved in 3′-end processing of histone mRNAs and maturation of telomerase, processing and assembly of RNAs as ribonucleoparticles like snRNPs, snoRNPs and sca-RNPs.

**Table 1.** Composition and function of different nuclear particles (Cajal bodies, PML bodies, PcG clusters, Insulator speckles, Nuclear speckles and Nucleolus).

Nuclear Particle	Composition	Functions	References
Cajal bodies	Coilin, Fibrillarin, SMN1, Gemins, Nap57, NO38, GAR1,NOPP140, TCAB1. RNAs *U85* scaRNA, *U87* scaRNA, *U88* scaRNA, *U89* scaRNA, *U90* scaRNA, *U91* scaRNA, *U92* scaRNA, *U2* snRNA, *U4* snRNA, *U5* snRNA, *U6* snRNA, *U3* snoRNA, *U8* snoRNA, *U14* snoRNA, *TERC RNA*	• Processing 3′ end of histonemRNA. • Involved in the maturation of telomerase. • Processing and assembly of ribonucleoproteins like snRNPS, snoRNPs and sca-RNPs. • Found to interact with gene like RNU1 and RNU2. • Involved in intra- and inter-chromosomal interactions of chr1, chr6 and chr17	^40^^,^^39^^,^^41^^,^^99^^,^^100^^,^^101^
Promyelotic leukemia (PML) bodies	PML,CBP, and other proteins such as Sp100, BLM, Daxx, Hipk2, Mdm2, p53, SUMO-1, TRF1, TRF2	• Involved in antivirus response, apoptosis, telomere shortening, DNA repair, cell cycle control etc. • Required for IFN-γ MHC II expression. • Also involved in gene repression with Daxx.	^58^^,^^29^^,^^30^^,^^79^^,^^80^^,^^102^^,^^103^
PcG clusters/bodies	Mostly components of PRC1 have been found in PcG clusters/bodies PH, Pc, RING1, CBXs and BMI1 PcG like proteins SOR-1 and SOP-2 proteins in *C.elegans.*	• Involved in genes silencing. • Involved in the compaction of HOX genes. • Maintain proper expression of HOX genes. • Mediates long-range genomic interactions. • A PcG protein, EZH2 helps in the the formation of loop within the GATA-4 locus.	^29^^,^^47-50^^,^^18^^,^^60^^,^^61^^,^ ^67^^,^^102^^,^^103^
Insulator bodies/speckles	CTCF (mammals), CP190, Su(Hw), mod(mdg4), BEAF, chromotor, dCTCF (Drosophila).	• Help in shaping chromatin topology, present at inter-TAD boundaries. • Involved in the formation of chromatin loops. • These may be involved in intra-TAD interactions.	^70^_,_^71^^,^^72^^,^^74^^,^^16^
Nuclear speckles	SR proteins (SF2/ASF, SC35, SRp20, SRp40, SRp55, SRp75, SRp30c, 9G8, and SRp54), CLK/STY, PRP4, PSKH1, eIF4Aiii and protein phosphatase 1. RNAs *U1* snRNA, *U2* snRNA, *MALAT1* RNA, Poly(A)^+^ RNA	• pre-mRNA splicing factors, including snRNPs and serine/arginine-rich (SR) proteins. • Regulates the post-translational modification of splicing factors. • Hubs for gene activation • Mediates interchromosomal interaction events induced by hormones.	^30^^,^^100^^,^^104^
Nucleolus	Nucleolin, B23, Fibrillarin. Transranslational factors and structural proteins including keratin, lamins and tubulin have also been identified in this compartment. RNAs *rRNAs, snoRNAs (U3* snoRNA, *U8* snoRNA, *U13* snoRNA, *U14* snoRNA, *U17* snoRNA, *E2* snoRNA, *E3* snoRNA), snRNAs, tRNAs, 7SL RNA.	• Primarily associated with ribosome biogenesis but it is also involved in several different functions like genome organization, stress response, cell cycle and proliferation. • intranuclear and nuclear–cytoplasmic transport, • modification and assembling of snRNAs • sequestrating proteins that control cell-cycle check points including Mdm2, Cdc14, and Pch2.	^98^^,^^105^^,^^106^

Several gene loci have been shown to interact with cajal bodies. RNU1 and RNU2 containing tandemly repeated snRNA genes were shown to interact with cajal bodies using immunostaining and FISH.^45^ RNU3 locus, containing clustered but not tandemly repeated U3 genes, was also shown to interact with cajal bodies.^46^ Analogous to rDNA sequences, which are called nucleolus organizers, cajal body interacting loci were called as “cajal body organizers." The histone locus that contains the cluster of histone genes, was also shown to interact with cajal bodies. These, studies suggested that gene loci having tandemly repeated or clustered genes interact with cajal bodies. However, loci like U4, U11 and U12, having single or 2 genes were also shown to interact with cajal bodies.^47^ Further studies investigating interaction of DNA loci with cajal bodies suggested that association of genomic loci with cajal bodies is transcription dependent.^45^^,^^48^^,^^49^ Interaction of U2 loci with cajal bodies was shown to be mediated by nascent pre-U2 RNA when transcription was going on.^50^ A recent study used a relatively high throughput and genome wide approach to investigate the organization of genomic regions around cajal bodies. Using a comprehensive FISH and 4C-seq analysis, authors showed that highly expressed genes including sn/snoRNA and histone genes, distributed throughout the genome clustered around the cajal body. 4C-seq analysis revealed intra- as well inter chromosomal interactions. An interaction hub was observed on chromosome 1. Chromosomes 6 and 17, harboring histone gene locus (HIST1) and RNU2, respectively were found to form the inter-chromosomal interaction with chromosome 1, and all 3 chromosomes associate with cajal body. Interestingly, upon perturbation of cajal bodies by knock down of their components, the chromosomal organization observed in wild type cells was dissolved, suggesting the role of cajal bodies in orchestrating the spatial organization of genome,^51^ with mostly active genes surrounding it.

### Polycomb clusters

Polycomb Group proteins (PcG) bodies/clusters are associated with silenced genes. PcG proteins, conserved from *Drosophila* to mammals and plants, are important for proper development of metazoan organisms. Polycomb proteins exist in the form of protein-protein complexes namely polycomb repressive complex 1 (PRC1), polycomb repressive complex 2 (PRC2) etc. For each of the *Drosophila* PcG protein there exist more than one homolog in mammals, increasing the diversity of PcG complexes. Initial immunostaining experiments of PcG proteins revealed few bright foci and diffused staining pattern. These foci were called as “Polycomb bodies," and were found in *Drosophila*, *C. elegans*, mouse cells, humans cell lines, and in cancerous cells as well.^52–55^ Using super-resolution microscopy we found that PcG proteins in *Drosophila* cells are distributed in the form of hundreds of nanoscale clusters varying in size from 10s to 100s of nanometers. This implies that PcG proteins are organized in the form of discrete clusters.^29^ The larger clusters might appear as “PcG bodies” and smaller more abundant clusters as diffused molecules when observed by confocal microscopy. However, it remains to be seen whether a similar nanoscale organization of PcG proteins exists in other organisms.

PcG foci/clusters like other nuclear bodies are membraneless dynamic subnuclear structures. Fluorescence Recovery After Photobleaching (FRAP) experiments have shown that PcG proteins exchange between PcG foci and surrounding molecules.^56^^,^^57^ Based on the recovery time during FRAP experiments, PcG proteins within PcG foci were shown to be of 3 different fractions; fast (2–8 s), slow (10–20s) and immobile within 300s, suggesting a heterogeneous association kinetics. We and others have shown that SAM-domain mediated polymerization of a PRC1 component, polyhomeotic (PH) is important for clustering of PcG proteins into nanoscale clusters and for larger foci in *Drosophila* and mouse, respectively .^29^^,^^58^ A microscopy based genome-wide RNAi screen identified 129 genes involved in modulating the distribution of PcG foci. This study showed that sumoylation of polycomb (PC) subunit of the PRC1 complex regulates the size of PcG clusters.^59^

Several studies have shown that non-coding RNAs associate with PcG proteins and can modulate their activity.^60–62^ Yang et al., showed that methylated Pc2 binds the TUG1 ncRNA and holds the target genes to PcG clusters but upon demethylation Pc2 binds the NEAT2/MALAT1 and target genes get relocated to interchromatin granules.^61^ A recent study showed that lncRNA, CAT7 copurifies with PRC1 and regulates its binding to MNX1 locus during early neuronal differentiation.^63^ In case of *Drosophila* it has been shown that RNAi machinery components colocalize with PcG bodies and are required for clustering of polycomb response elements (PREs).^64^ Although, these studies suggest ncRNAs can be components of PcG clusters, further studies are needed to show that like other nuclear bodies such as cajal and PML bodies, ncRNAs are bonafide structural and functional components of PcG clusters.

PcG proteins maintain proper expression of Hox genes throughout the development of *Drosophila*. Immuno-fluorescence in situ hybridization (FISH) experiments demonstrated that PREs of Hox genes localize to PcG clusters when the genes are silenced.^65^^,^^66^ Fab7 PRE controlling the expression of Hox gene AbdB in *Drosophila*, localizes to the PcG foci in the head where AbdB is repressed but not in the posterior regions where AbdB is expressed, suggesting the silencing nature of PcG clusters.^65^ Further immuno-FISH experiments revealed that intensity of the PcG foci correlates with the size of underlying genomic region, for example, BX-C which is about 350 kb localizes to more intense PcG cluster than NK-C which is about 200 kb. This was further substantiated by the observation that the paired Hox gene loci of homologous chromosomes are present in more intense PcG foci than in unpaired condition.^55^ FISH experiments also demonstrated that long-range kissing interaction between ANTP-C and BX-C separated by about 12 Mb is dependent on the PcG binding regulatory regions of these loci. Recently, a high resolution FISH study showed that compaction of a Hox gene complex depends on PRC1 through knockdown of the PH subunit that resulted in decompaction of the Hox gene cluster.^18^ In addition to FISH, electron microscopy studies also showed that PcG clusters detected by immunolabeled BMI1 are enriched in condensed chromatin throughout the nuclei of U2-OS cells.^67^ Electron microscopy based studies also showed that PRC1 or its subunits from different organisms compact nucleosomal arrays *in vitro*.^68–70^ Lau et al., showed that mutation in the nucleosome compaction region of the Cbx2 PcG protein leads to homeotic transformations in mouse.^71^ From all these microscopy based studies it is clear that PcG clusters/foci are sites of highly condensed and compacted chromatin.

Besides microcopy studies, chromosome conformation capture based studies have also unraveled the role of PcG proteins in mediating/stabilizing genomic interactions in *Drosophila* and in mammals. 3C and 4C based studies from different laboratories showed that polycomb response elements (PREs) in *Drosophila* interact and cluster together in a repressed state, which is dependent on PcG proteins.^65^^,^^66^ Similarly looping within the GATA-4 locus was shown to be dependent on the PcG protein, EZH2.^72^ In case of mouse ESCs, PRC1 was shown to act as an important regulator of genome architecture. PRC1 organizes the genomic interaction networks of 4 Hox gene clusters and early developmental genes. These interaction networks were perturbed upon depletion of the RING1B component of PRC1.^73^ By using 4C-seq we unraveled multiscale interactions within BX-C and between BX-C and rest of the chromosome 3R of *Drosophila*. These genomic interactions were reduced upon mutations in the SAM domain of the PRC1 subunit, PH, and the same mutations disrupted the clustering of PcG proteins into hundreds of nanometer scale clusters.^29^ From our and other studies it seems that PcG proteins bound to chromatin self-associate into hundreds of clusters by different mechanisms like SAM domain polymerization of PH, sumoylation of polycomb (Pc) and ncRNAs, and mediate chromatin interactions at multiple genomic and spatial scales.

### Insulator speckles

Another important group of proteins involved in mediating genomic interactions are insulator proteins. Insulator proteins appear as immunofluorescent foci and were called as “insulator bodies” in *Drosophila* cells.^74^ These proteins are bound to insulator elements and prevent the spreading of transcription/repression along the genome.^75^ In case of mammals CTCF is the only insulator protein but in *Drosophila* there are several of them like CP190, Su(Hw), mod(mdg4), BEAF, chromotor etc. in addition to dCTCF. These proteins were found to form about 10–20 “insulator bodies” within diploid nucleus of *Drosophila* cells, and were reported as clusters of insulators held together by insulator proteins, and thus acting as hubs of genomic interactions.^76^^,^^77^ However, a latter study reported that detection of bright “insulator bodies” depends on procedures followed for dissection or staining like salt concentrations in buffers and time taken. Treatment with high NaCl concentration and longer time incubations in phosphate buffered saline (PBS) resulted in detection of insulator bodies. These appeared as aggregates of insulator proteins, devoid of insulator elements and got dissolved when cells were put back in isotonic conditions.^78^ A few other studies also supported this idea that “insulator bodies” are aggregates of only insulator proteins and not of underlying insulator elements.^79^

A recent study taking advantage of high-resolution structured illumination microscopy (SIM) detected about 100 speckles of dCTCF and CP190 proteins per nucleus, in contrast to 10–20 bright foci when lower resolution microscope was used for imaging. To find out the role of these insulator speckles in genomic interactions, the authors analyzed the proximity of these insulator speckles with kissing interactions of Hox gene complexes separated by about 12 Mb of genomic distance on Chr3R of *Drosophila*. The authors reported that dCTCF speckles are significantly closer to interacting Hox genes than non-interacting genomic loci.^28^ However, the interaction frequency of Hox gene clusters was very low. Two earlier studies supporting this observation reported that insulator elements and not PcG response elements are responsible for long-range interactions among PcG target genes and targeting of PcG target genes to different subnuclear compartments depends upon their state of expression.^80^

A lot of literature exists on the role of insulator proteins in shaping chromatin topology and has been reviewed before.^75^ Insulator proteins are bound at thousands of sites along the genome. They are involved in formation of chromatin loops, whose size depends upon the number and combination of insulator proteins and cohesion bound at the bottom of the loop.^17^ Insulator proteins particularly CTCF has been found at inter-TAD boundaries, which are sites of low frequency local interactions, and the strength of boundary has been shown to correlate with binding of insulator proteins.^81^ Furthermore, recent studies showed that directionality of CTCF binding sites in mammalian cells determines the formation of loops and TADs.^82^ However, insulator proteins are not present only at boundaries but also within the TADs and might be involved in mediating intra-TAD interactions. From these studies it clear that insulator proteins play an important role in shaping the genome architecture but the nature of insulator bodies/speckles and their role in 3D genome organization remains ambiguous.

### Other nuclear bodies

Promyelocytic Leukemia (PML) bodies are detected by immunostaining of their PML protein and are usually spherical in shape and vary in diameter from 0.2–1 μm and in number from about 5–30 per nucleus.^83^ They are involved in number of functions like antivirus response, apoptosis, telomere shortening, DNA repair, cell cycle control etc.^30^^,^^31^ Given their multifunctional nature their composition varies and about 166 proteins have been shown to be associated with PML bodies (Table 1). A large number of these proteins are involved in transcription regulation. Several studies reported that transcriptionally active genomic loci are associated with PML bodies. Ulbricht et al., showed that IFN-γ treatment increases spatial proximity of MHC II gene cluster with PML bodies and PML protein is required for IFN-γ MHC II expression.^84^ However, another study points to the role of PML bodies in gene repression. Daxx, a transcriptional corepressor is a key component of PML bodies and gets SUMOylated on its C-terminus. SUMOylation of Daxx is important for both association of Daxx with the PML protein as well as for targeting of Daxx to promoters of anti-apoptotic genes.^85^

Nuclear speckles are ribonucleo-protein macromolecular assemblies enriched in splicing factors and also contain proteins like ser2-phosphorylated RNA pol II.^86^^,^^87^ Many actively transcribing genes have been shown to localize in close proximity of nuclear speckles making them as hubs of active genes. Association of Hsp70 gene with nuclear speckles has been shown to be mediated by its promoter and is dependent on transcription.^88^ Nevertheless, the association does not correlate with level of transcript and Hsp70 does not have introns. Further studies showed that directed motion of Hsp70 in response to heat shock is actin dependent, as actin depolymerization blocked the association of Hsp70 with nuclear speckles.^89^

Apart from above discussed subnuclear clusters/foci/bodies, nucleolus is the most prominent subnuclear body. It is organized around tandemly repeated rDNA gene clusters known as nucleolus organizer regions (NORs). The nucleolus has been primarily associated with ribosome biogenesis but it is also involved in several different functions like genome organization, stress response, cell cycle and proliferation.^30^^,^^31^ Although it is a site of active transcription at the center, at its periphery it is associated and surrounded by silent heterochromatin.^90^ The inactive X chromosome, imprinted genes and centromeres are localized to the nucleolar periphery.^91^^,^^92^ A couple of independent studies investigated the nucleolus associated genomic regions using deep sequencing.^93^^,^^94^ One of the studies identified nucleolus associated chromatin domains containing one thousand 37 genes. A genome-wide analysis of nucleous associated domains (NADs) in human cells showed that these domains are characterized by low gene density and transcriptionally repressed genes, suggesting that these might be general properties of chromatin associated with nucleolus.^94^ However, in case of yeast RNA pol III actively transcribed genes are tethered to the nucleolus but nearby RNA pol II transcribed genes, also associated with nucleolus, are repressed.^95^^,^^96^ Different factors like RNAs, proteins and post-translational modifications of histones have been reported to be involved in tethering of different genomic loci to nucleous. ncRNAs like Xist and Kcnq1ot have been shown to be involved in mediating tethering of inactive X-chromosome and imprinted Kcnq locus to nucleolar periphery. Proteins like CTCF, modulo, nucleoplasmin and histone modifications like H3K9me are involved in sequestering the NADs to the nucleolus.^90^

### Conclusion and future perspectives

**Figure 4.**
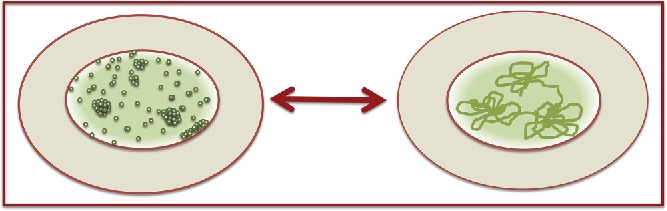
Relationship between subnuclear distribution of proteins and genome organization: Sub-nuclear distribution of proteins (left) can regulate 3D genome organization (right); however, it is also possible that spatial arrangement of genome can affect distribution of proteins within the nucleus.

In this review we discussed connections between subnuclear distribution of proteins and organization of genome within the nuclear space. From above discussed literature it is clear that spatial distribution of proteins within the nucleus plays an important role in shaping the genome architecture (Fig 4). We first classified distribution of proteins into the diffused and aggregated fractions based mainly on images available from confocal or other lower resolution microscopy studies. However, from super-resolution microscopy it appears that the fraction of a protein that appears as diffused at lower resolution might be aggregated/clustered into nanoscale speckles or clusters. Hence, in future it seems important to use super-resolution microcopy to fully understand the subnuclear distribution of proteins and then link it with genome organization.

Although polymer modeling provides 3D models of genome organization,^97^^,^^98^ a direct experimental picture of how DNA and associated proteins are arranged with respect to each other in 3D nuclear space is not available. Excavating the 3D organization of genome along with associated proteins will be challenge for future.

More directed studies analyzing genome-wide interactions between genome and subnuclear body/clusters need to be performed to identify the nuclear body/cluster associated domains to understand the relationship between the organization of proteins and genome organization. Furthermore, studies unraveling mechanisms which orchestrate the interactions between different components of subnuclear clusters (Protein-DNA, protein-protein, protein-RNA and DNA-RNA) will be highly valuable to understand the determinants of genome organization.

ChIP-seq studies have provided enormous data about binding of thousands of proteins along the genome and this one-dimensional arrangement of proteins correlates with 3D organization of chromatin. It might be helpful to integrate the spatial distribution of these proteins along with their one-dimensional binding information while interpreting their role in genome organization.

## Abbreviations

TADs: Topologically associating domains
CTs: Chromosome territories
FISH: Fluorescence in situ hybridization
FRAP: Fluorescence recovery after photobleaching
PML: Promyelotic leukemia
HLBs: Histone locus bodies
PcG: Polycomb Group
PRC1: Polycomb repressive complex 1
SAM: Sterile α motif
CTCF: CCCTC-binding factor
CP190: Centrosomal protein 190
NADs: Nucleous associated domains
PPIN: Protein-protein interaction network.
